# A commentary on ‘Prediction models of surgical site infection after gastrointestinal surgery: a nationwide prospective cohort study’

**DOI:** 10.1097/JS9.0000000000001088

**Published:** 2024-01-17

**Authors:** Siqin Wang, Wenyan Liu, Limei Zhan, Yongchao He, Jing Xu

**Affiliations:** a Operating Room; b Disinfection Supply Center, Yantai Yuhuangding Hospital, Yantai, Shandong, People’s Republic of China

*Dear Editor*,

Surgical site infection (SSI) is a type of infection that is associated with surgery that usually appears at or close to the surgical incision within 30 days after the procedure^1^. It raises the risk of mortality and readmission and is frequently discovered following gastrointestinal surgery^2^. Yang *et al*.^3^ conducted an observational, multicenter, prospective cohort study analysis on patients who underwent gastrointestinal surgery. It appears that surgeons can lower the risk of SSIs by enhancing SSI prevention and health precautions for patients with chronic liver and renal illness, colon surgery, open surgery, and colostomy/ileostomy at the outset of the procedure.

In this study, 17 535 gastrointestinal procedures performed between 2021 and 2022 across 57 facilities were analyzed. With adequate prediction accuracy and therapeutic advantages for postoperative SSI risk in patients after gastrointestinal surgery, this model encompasses 20 predictive parameters, including baseline patient characteristics and perioperative care. According to the results of the study, among gastrointestinal procedures, colon surgery carries the highest risk of SSI. Furthermore, the study demonstrated that combining oral antibiotics (OA) with mechanical bowel preparation (MBP) was a more effective preoperative bowel preparation strategy than either strategy alone in lowering the incidence of SSI. According to the aforementioned research, patients undergoing colorectal surgery should be treated with routine combination bowel preparations (MBP and OA) as standard therapy. Thus, it is desirable to include this in the models of hospital best practices.

However, there were still a lot of restrictions. First, some residual confounding factors may not have been considered. When analyzed with SHAP (SHapley Additive exPlanations), several research found that organ-space SSI present at the time of surgery, operative time, oral antibiotic bowel prep, surgical technique, procedure CPT (Current Procedural Terminology) code, body mass index, ASA (American Society of Anesthesiologists) classification, and age had the most impact on model decision-making^4^. The model’s decision-making was influenced by preoperative care measures such as hair trimming, OA, MBP, antiseptic wash the night before and the day of the operation, antibiotic prophylaxis, and more. Postoperative care measures such as wound care, particularly for patients with colostomies, influenced model decision-making.

Second, it is preferable to investigate the prognosis of superficial, deep, and organ-space SSI separately. Another way to characterize SSI is to have organ-space, deep, or superficial SSI. Skin and subcutaneous tissue are involved in superficial SSI; muscle and fascia are involved in deep SSI, and the anatomy that was altered during the treatment is involved in organ-space SSI^5^. Ultimately, the variables gathered in the database, the precision of data collection, the 30-day interval, missing data, the kind of systemic antibiotic prophylaxis, wound care (closed or left open), hospital type, and hospital volume all restrict the predictive power of the models.

The purpose of this study was to investigate the variables that affect the likelihood of SSI incidence and to develop a clinical prediction model that will offer useful instruments and assessment techniques for SSI prevention and early intervention. This large-scale observational, multicenter, prospective cohort study helps with clinical trial design and decision-making by providing a model for predicting SSI risk. This innovative strategy offers insightful direction and motivation for the ongoing advancement of this field of study. Further research could use similar computer technology to constantly update these models with new data and deliver real-time, tailored risk prediction.

## Ethical approval

This manuscript is a comment. Don’t need ethical approval.

## Consent

This manuscript is a comment. Don’t need patients’ consent.

## Sources of funding

None.

## Author contribution

S.W. and W.L.: study concept or design, data collection, data analysis or interpretation, and writing the paper; L.Z. and Y.H.: data collection; J.X.: study concept or design and writing and revising the paper.

## Conflicts of interest disclosure

This manuscript is a comment without conflicts of interest.

## Research registration unique identifying number (UIN)

This manuscript is a comment. Don’t need UIN.

## Guarantor

Jing Xu.

## Data statement

This manuscript is a comment. Don’t need a Data availability statement. However, all the data from the current study are publicly available.

## Provenance and peer review

This manuscript is a comment without being invited.
